# CircTEC Inhibits the Follicular Atresia in Buffalo (*Bubalus bubalis*) via Targeting miR-144-5p/FZD3 Signaling Axis

**DOI:** 10.3390/ijms26010153

**Published:** 2024-12-27

**Authors:** Juanru Cheng, Qinghua Xing, Yu Pan, Yanyan Yang, Ruimen Zhang, Deshun Shi, Yanfei Deng

**Affiliations:** 1 Guangxi Key Laboratory of Animal Breeding and Disease Control, College of Animal Science and Technology, Guangxi University, Nanning 530004, China; 2118401014@st.gxu.edu.cn (J.C.); whd99fly@163.com (Q.X.); 2218401005@st.gxu.edu.cn (Y.Y.); 20230099@gxu.edu.cn (R.Z.); 2 Chongqing Academy of Animal Sciences, Chongqing 402460, China; cqaa-pan95@outlook.com

**Keywords:** follicular atresia, buffalo, CircRNA, miRNA, post-transcriptional regulation

## Abstract

The specific expression profile and function of circular RNA (circRNA) in follicular atresia remain largely unknown. Here, the circRNA expression profiles of granulosa cells derived from healthy follicles (HFs) and antral follicles (AFs) in buffalo were analyzed by RNA-seq, and the mechanism of a differentially expressed circRNA (DEcircRNA) circTEC regulating the granulosa cell function that affects follicular atresia was further explored. RNA-seq results showed that a total of 112 DEcircRNAs were identified. Among them, circTEC was highly expressed in HF, and its circular structure was confirmed by RNase R digestion assay, reversed PCR and Sanger sequencing. Functional experiments demonstrated that circTEC promotes the proliferation and steroid hormone synthesis of buffalo granulosa cells (bGCs), and it also inhibits their apoptosis. In-depth mechanism analysis showed that the expression level of circTEC in bGCs from AFs was adversely related to miR-144-5p and consistent with FZD3. CircTEC acts as an endogenous sponge of miR-144-5p to regulate the expression of the target gene FZD3 in AFs, which promotes the proliferation of bGCs and inhibits bGCs apoptosis, thereby inhibiting follicular atresia in buffalo. In summary, our study revealed the regulatory role of the circTEC/miR-144-5p/FZD3 axis during follicular atresia in buffalo. These results provided new insights into the biological mechanism underlying follicular atresia.

## 1. Introduction

The mammalian ovary contains millions of primordial follicles during the fetal period, but only a handful of follicles develop to ovulation. More than 99% of follicles undergo a degenerative process called “follicular atresia” [1]. Follicular atresia maintains the stability of the mammalian ovary via continuously liminating follicles, but it also results in a large number of follicle resources to be wasted. So, methods to develop and utilize the abundant resources of ovarian follicles are consistently sought in animal reproduction research [2]. Chinese buffalo (*Bubalus bubalis carabanesis*) is an important domestic animal distributed in the tropical and subtropical regions of China. However, the reproductive efficiency of buffaloes is generally lower than that of cattle due to multiple factors, such as poor estrus manifestation, low ovarian reserve and high incidence of atresia [3]. Therefore, elucidating the molecular mechanism of buffalo follicle atresia will be expected to reverse the fate of follicles, thereby effectively utilizing more follicle resources and improving its reproductive efficiency.

Circular RNA (circRNA) is a new type of ncRNA produced from the precursor mRNA back-splicing of gene exons in eukaryotes, and it has been proven to act as another class of important modulators of different biological processes and diseases [4]. CircRNA is resistant to exonuclease-mediated degradation due to its covalently closed-loop structure, thus making it more stable with longer half-lives compared to other types of RNA. In mammals, circRNA has tissue specificity and expression specificity as well as a high conservatism of evolution [5]. Several studies have indicated that circRNA regulates gene expression at both the transcriptional and post-transcriptional level through a variety of regulatory mechanisms such as interacting with RNA-binding protein, acting as sponges for miRNA, and interfering with pre-mRNA processing. Additionally, a few endogenous circRNAs can translate as functional proteins, and some others can act as sources of pseudogene derivation. At present, the use of circRNAs as an miRNA sponge to regulate the expression level of targeted gene is the most widely studied function of circRNAs [4,6,7].

Based on advanced sequencing technology, abundant circRNAs have been discovered in the ovary of human [8,9], mouse [10], pig [11], bovine [12] and goat [13] species, and some of these circRNAs have been verified to play an important regulatory role in various reproductive physiological or pathological processes, such as ovarian cancer [14,15], polycystic ovary syndrome [16], ovarian aging [17] and follicle development [18]. A genome-wide deep circRNA sequencing-based study firstly revealed 192 differentially expressed circRNAs (DEcircRNAs) in healthy and early atretic antral follicles in pigs [19], and further studies demonstrated the regulatory role of the DEcircRNAs in granulosa cells as miRNA sponges on connective tissue growth factor (CTGF) [20], inhibin-activin balance [21], and serine and arginine rich splicing factor 1 (SRSF1) [22]. In addition, Meng et al. also revealed the expression profiles of circRNAs in porcine granulosa cells derived from healthy and atretic antral follicles and identified 62 DEcircRNAs, discovering that they mainly act on oxidative stress and cell apoptosis pathways [23]. So, accumulating evidence has suggested that circRNA may play important regulatory functions during follicle atresia. Our early whole-transcriptome profiling studies revealed the molecular mechanisms involved in the atresia of buffalo follicles from the aspect of mRNA, miRNA and lncRNA [24]. However, the expression changes, function and regulatory mechanisms of circRNAs during the atresia process in buffalo remain to be revealed.

The normal follicular granulosa cell function is an important factor in the regulation of follicular atresia. This study was intended to reveal the circRNA expression profiles of granulosa cells derived from healthy and atretic antral follicles in buffalo by RNA-seq. And we found that a novel circRNA, circ-002750 (named circTEC), was significantly down-regulated in atretic follicles. The role and mechanism of circTEC in regulating the function of buffalo granulosa cells (bGCs) and affecting follicular atresia were further investigated. It is expected that this study will enrich the mechanism of circRNA in the field of reproduction and provide new clues for regulating follicular development to improve reproductive efficiency in domestic animals.

## 2. Results

### 2.1. Overview of circRNAs Expression Profiles in bGCs from Healthy and Atretic Follicles

**Figure 1:**
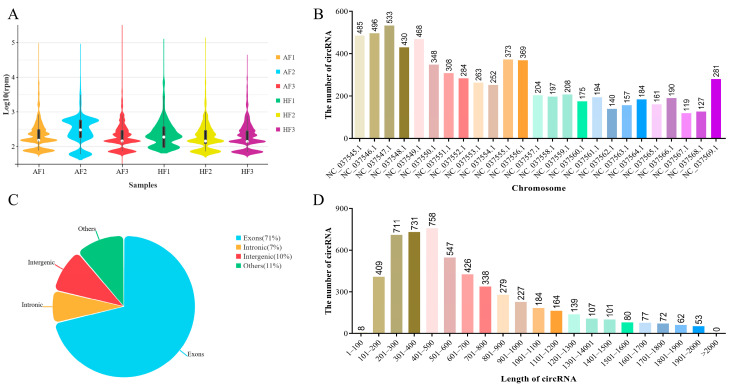
Overview of the identified circRNAs in bGCs from healthy and atretic follicles. (**A**) Boxplot shows the overall expression levels of circRNAs in each sample. AF1, AF2, and AF3 were the granulosa cells in atretic follicles, and HF1, HF2, and HF3 were the granulosa cells in healthy follicles. (**B**) Distribution of all identified circRNAs in different chromosomes. (**C**,**D**) are the location and length distribution of all identified circRNAs.

In order to study the potential regulatory mechanism of circRNAs that affects follicular atresia in buffalo, we profiled the expression of circRNAs in bGCs obtained from healthy and atretic antral follicles by removing the ribosomal RNA RNA-Seq. The number of raw reads (on average) in HFs and AFs was 60,065,290 and 44,166,709, respectively. The number of clean reads (on average) was 59,527,143 and 43,777,092, respectively. The average rate aligned to the *Bubalus bubalis* genome (Bubalus_bubalis_Jaffrabadi_v3.0. https://www.ncbi.nlm.nih.gov/datasets/genome/GCA_000180995.3/, accessed on 29 June 2019) was about 88% (Supplementary Material S3: Table S5). Based on the circRNAs identification standard (7), we defined circRNAs as containing at least two unique back-spliced reads. A total of 6443 circRNAs were identified after mapping to the reference sequence (Supplementary Material S3: Table S6), which originated from 3001 genes (Supplementary Material S3: Table S7). The difference in circRNA expression was minimal among the samples (Figure 1A). CircRNAs were widely distributed across all chromosomes, of which chromosome 3 containing the largest number of circular transcripts with 533 circRNAs (Figure 1B). Most circRNAs derived from spliced exons, whereas smaller fractions aligned with introns, unannotated regions of the genome and antisense regions to known transcripts (Figure 1C). The length of all circRNAs was less than 2000 nucleotides (nt), and the mean length was approximately 700 nt (Figure 1D).

### 2.2. Identification and Analysis of DEcircRNAs

**Figure 2:**
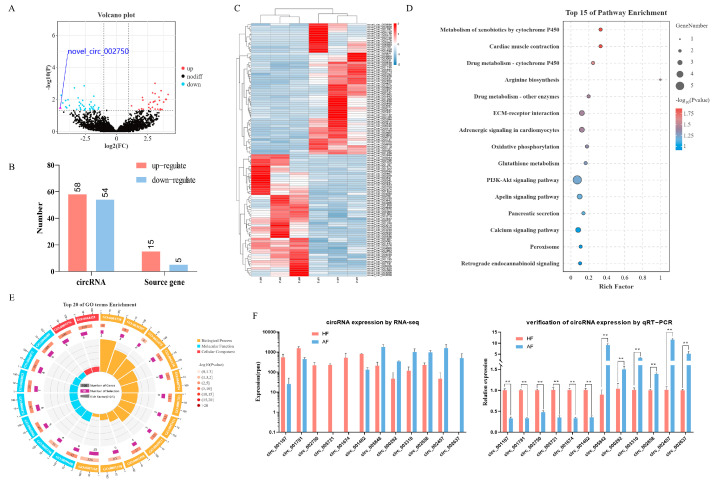
Identification and analysis of DEcircRNAs in granulosa cells during buffalo follicular atresia. (**A**,**C**) are the volcano plot and clustering heatmap of DEcircRNAs between healthy and atretic follicles, respectively (in the volcano plot, red for up-regulation and blue for down-regulation). (**B**) Statistics of the number of DEcircRNAs and their host genes. (**D**) and (**E**) are the KEGG and GO enrichment analysis of DEcircRNAs host genes, respectively (GO enrichment analysis includes biological processes, molecular functions, and cellular components). (**F**) Validation of randomly selected 12 DEcircRNAs (6 up-regulated, 6 down-regulated) by RT-qPCR. ** *p*  <  0.01.

The analysis of circRNAs expression showed that a total of 112 DEcircRNAs (fold change ≥ 2 and *p* ≤ 0.05) were identified (Supplementary Material S3: Table S8), of which 58 circRNAs were significantly up-regulated, and 54 circRNAs were significantly down-regulated in AFs as compared to HFs (Figure 2A,B). Their heatmap showed that there was an obvious separation between the HF and AF groups (Figure 2C). In order to analyze whether the expression change of circRNAs was consistent with that of host genes, we directly compared the up-regulation and down-regulation of DEcircRNAs and differentially expressed mRNAs from those of the circRNA host gene. The results discovered that the expression patterns of DEcircRNAs were not consistent with the changes in host genes (Figure 2B). This suggests that the difference in DEcircRNAs expression is difficult to explain by the variation in host genes expression. To verify the accuracy of RNA-seq data, 12 DEcircRNAs were randomly selected for quantification by RT-qPCR, and the results were consistent with the RNA-seq data (Figure 2F). In addition, to further explore the functional relationship between DEcircRNAs and follicular atresia, gene ontology (GO) and Kyoto Encyclopedia of Genes and Genomes (KEGG) analysis were carried out for host genes of DEcircRNAs by OmicShare tools (https://www.omicshare.com/, accessed on 29 June 2019). The enriched GO terms (including the biological processes, cellular components, and molecular functions) are shown in Figure 2E and Supplementary Material S3: Table S9. The most significantly enriched GO terms are Receptor activity (GO:0004872), Transmembrane receptor activity (GO:0099600), and Morphogenesis of a branching epithelium (GO:0061138). KEGG analysis revealed that DEcircRNAs host genes were mainly enriched in Metabolism of xenobiotics by cytochrome P450, Cardiac muscle contraction, Drug metabolism—cytochrome P450, etc. (Figure 2D; Supplementary Material S3: Table S10).

### 2.3. Characteristics of circTEC in bGCs

**Figure 3:**
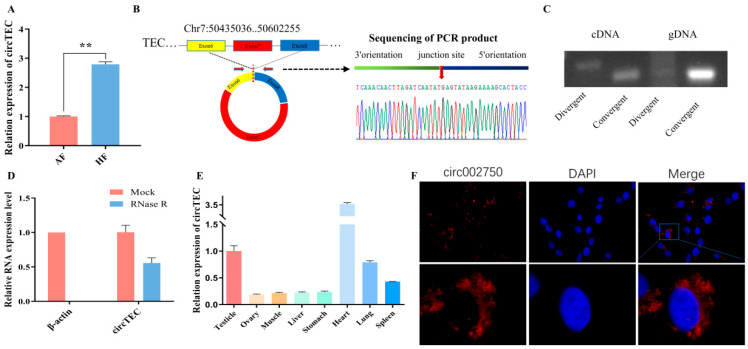
Characteristics of circTEC in bGCs. (**A**) Expression changes of circTEC in bGCs from HF and AF. (**B**) Sketch of the structure of circTEC, which is generated from the six to eight exons of the TEC gene via back-splicing. The back-splicing site of circTEC was validated by Sanger sequencing. (**C**) Divergent primers and convergent primers were designed and synthesized for circTEC, and PCR amplification reactions were performed using cDNA and gDNA as templates. The agarose gel electrophoresis of the PCR products shown in (**C**). (**D**) circTEC expression in bGCs with and without RNase R digestion. β-actin served as a linear RNA control. (**E**) The expression levels of circTEC in different tissues of embryonic buffalo. (**F**) The subcellular localization of circTEC was detected by FISH assay. circTEC was labeled with red fluorescence, and the cell nucleus was stained by DAPI (blue fluorescence). Magnification 63×, ** *p*  <  0.01.

In transcriptome sequencing data, the expression level of circ-002750 in HF was found to be significantly higher than that in AF (Figure 2A). Moreover, a significant decrease in circ-002750 was detected in AFs relative to HFs through RT-qPCR (Figure 3A). Circ-002750, located on chromosome 7, is 286 bp in length and is back-spliced by six to eight exons of the TEC gene, which we renamed circTEC. Sanger sequencing of the PCR products amplified by specific divergent primers further confirmed the back-splice junction site of circTEC (Figure 3B). Both divergent and convergent primers were used to amplify circTEC. The agarose gel electrophoresis results showed that circTEC could only be amplified from cDNA by divergent primers (Figure 3C). We then investigated the stability of circTEC in bGCs. RNase R digestion assays showed a dramatically reduced ACTIN expression level, and circTEC was resistant to RNase R, demonstrating that circTEC was structurally stable (Figure 3D). In addition, qPCR analysis of tissue expression showed that circTEC was expressed in various tissues, such as ovary, heart, and testicle (Figure 3E). Fluorescence in situ hybridization (FISH) assays showed that circTEC was mainly localized to the cytoplasm in bGCs (Figure 3F). Taken together, these results revealed that the existence and circular structure of circTEC might play an important role in buffalo follicular atresia.

### 2.4. Effects of circTEC on Apoptosis, Proliferation and Steroid Hormone Synthesis of bGCs

**Figure 4:**
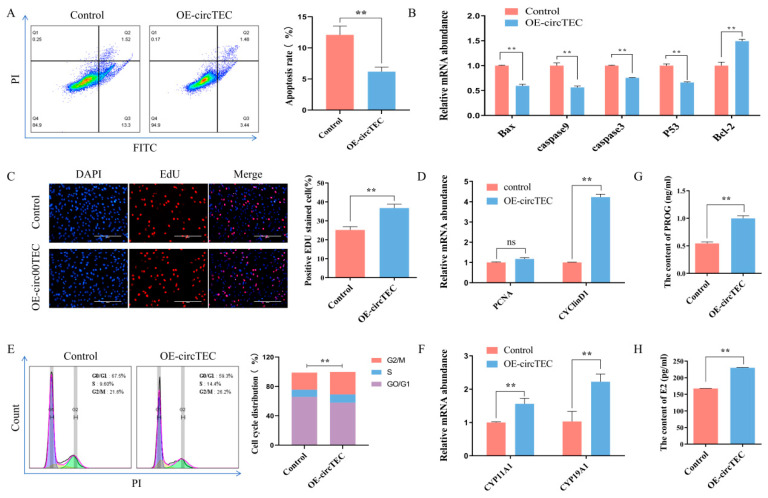
Effects of circTEC on functions of bGCs. bGCs were transfected with pK25-circTEC for 24 h and then apoptosis, proliferation and steroid hormone synthesis were assessed. (**A**) Cell apoptosis of bGCs was determined and counted by Annexin V-FITC/PI double staining followed by flow cytometry. The Q2 and Q3 quadrants represent apoptosis cells. (**B**) The mRNA expression of marker genes (*Caspase3*, *Bcl2*, *Bax*, etc.) of cell apoptosis was measured by RT-qPCR. (**C**) Cell proliferation of bGCs was determined and counted by 5-ethynyl-20-deoxyuridine (EdU) assays. Red for EdU and blue for DAPI. (**D**) The mRNA expression of cell proliferation marker genes (*PCNA* and *cyclin D1*) was measured by RT-qPCR. (**E**) Cell cycle of bGCs was determined and counted by propidium iodide binding followed by flow cytometry. (**G**,**H**) The concentrations of PROG (**G**) and E2 (**H**) in cell culture media were detected using ELISA. (**F**) The mRNA expression of steroid hormone synthesis key genes (*CYP11A1* and *CYP19A1*) by RT-qPCR. ns indicates no significant difference. ** *p*  <  0.01. Scale bars: 200 μm.

To explore the possible function of circTEC on bGCs, we transfected pK25-circTEC into bGCs to overexpress circTEC significantly (Figure S1). Then, flow cytometry, EdU, Elisa, WB and RT-qPCR assays were used to assess its function. First, the results of flow cytometry showed that the overexpression of circTEC significantly decreased the apoptosis rate of bGCs (Figure 4A). The key genes of cell apoptosis such as *Caspase3*, *Bcl2, Bax,* etc. were measured by RT-qPCR. It was found that pK25-circTEC inhibited the expression of *Bax*, *Caspase3*, *Caspase9* and *P53* at the mRNA levels, and it promoted the mRNA expression of *Bcl2* (Figure 4B). Secondly, EdU assay, cell cycle analysis and RT-qPCR were used to determine the role of circTEC in the cell proliferation of bGCs. EdU staining analysis showed that the overexpression of circTEC increased the proportion of EdU-positive cells (Figure 4C). Cell cycle analysis found that circTEC overexpression increased the proportion of bGCs in the S- and G2/M-phases, and it decreased the number of G0/G1 phase cells (Figure 4E). RT-qPCR analysis discovered that the overexpression of circTEC significantly promoted the mRNA expression levels of cell proliferation-related genes such as *PCNA* and *cyclin D1* (Figure 4D). Finally, the concentrations of E2 and PROG in the medium of bGCs after transfection with pK25-circTEC were detected by ELISA. The results revealed that the secretion of E2 and PROG in bGCs could be significantly promoted by circTEC (Figure 4G,H). We also determined the effect of circTEC on the expression of steroid hormone synthesis-related genes and found that it significantly increased the mRNA expression level of *CYP11A1* and *CYP19A1* (Figure 4F). Collectively, these results suggest that circTEC promotes the proliferation and steroid hormone synthesis of bGCs, and it also inhibits bGCs apoptosis.

### 2.5. CircTEC Regulated Proliferation and Apoptosis of bGCs via Serves as a Sponge for miR-144-5p

**Figure 5:**
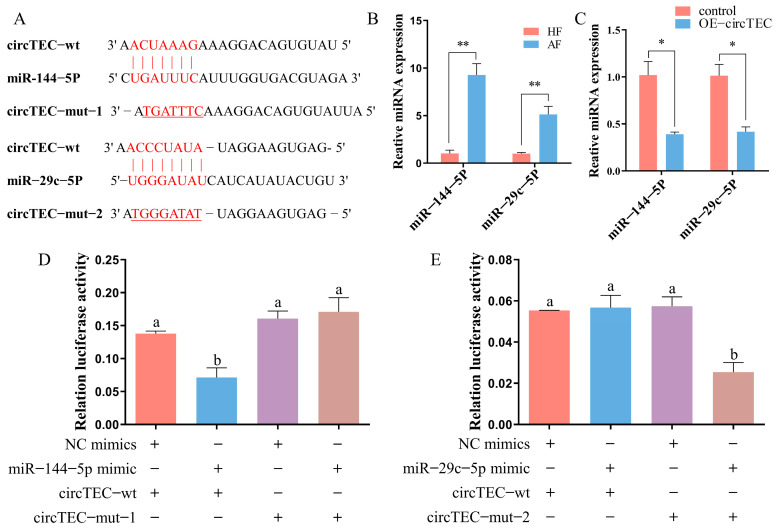
Direct interaction between circTEC and miR-144-5p. (**A**) Bioinformatics analysis predicted the binding sites between circTEC and miR-144-5p/miR-29c-5p, and the corresponding mutation vector targeting the binding sequences was designed. (**B**) Expression changes of miR-144-5p and miR-29c-5p in bGCs from HFs and AFs. (**C**) Expressions of miR-144-5p and miR-29c-5p in bGCs were measured by RT-qPCR after the overexpression of circTEC. (**D**,**E**) bGCs were co-transfected with miR-144-5p mimics (**D**) or miR-29c-5p mimics (**E**), and the reporter vector carrying wild-type or mutated corresponding binding site, and then luciferase activity was determined. The same letter denotes no significant difference (*p*  >  0.05), while different letters indicate a mean significant difference (*p*  <  0.05). * *p*  <  0.05, ** *p*  <  0.01.

Given that circTEC was mainly localized to the cytoplasm in bGCs (Figure 3F), we next investigated whether circTEC might act as an miRNA sponge to regulate gene expression. According to the results of bioinformatic analysis (http://www.targetscan.org/vert_72/, accessed on 2 October 2019), we identified two candidate miRNAs (miR-144-5p and miR-29c-5p) that may bind to circTEC (Figure 5A). RT-qPCR verified that the expression levels of miR-144-5p and miR-29c-5p were significantly higher in AFs than in HFs, which was contrary to the expression changes of circTEC during follicular atresia (Figure 5B). To further confirm the correlation between circTEC and candidate miRNAs, we enhanced the expression of circTEC in bGCs. The RT-qPCR assay results showed that the overexpression of circTEC significantly decreased the abundance of miR-144-5p and miR-29c-5p (Figure 5C). In addition, the entire circTEC sequence was inserted into the pmiRGLO luciferase reporter vector (pCK-circTEC-WT) and its mutation vector (pCK-circTEC-MUT) targeting the binding sequences of miR-144-5p and miR-29c-5p was constructed, respectively (Figure 5A). The introduction of miR-144-5p (but not miR-29c-5p) significantly inhibited the activity of pCK-circTEC-WT but had no difference effect on pCK-circTEC-MUT (Figure 5D,E). These results suggest that circTEC acts as a sponge for miR-144-5p but not miR-29c-5p.

**Figure 6:**
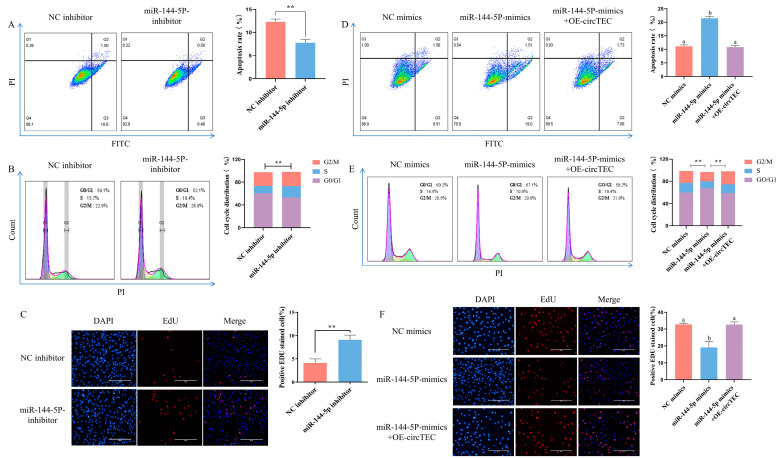
circTEC regulated proliferation and apoptosis of bGCs by acting as a ceRNA of miR-144-5p. (**A**) The apoptosis rate of bGCs was determined and counted by Annexin V-FITC/PI double staining followed by flow cytometry after the transfection of miR-144-5p inhibitor. The Q2 and Q3 quadrants represent apoptosis cells. (**B**) Cell cycle of bGCs was detected and counted by PI binding followed by flow cytometry after the transfection of miR-144-5p inhibitor. (**C**) Cell proliferation of bGCs was measured and counted by EdU assays after the transfection of miR-144-5p inhibitor. Red for EdU and blue for DAPI. (**D**) bGCs were transfected with pK25-circTEC and/or miR-144-5p mimic, and then cell apoptosis was determined and counted by Annexin V-FITC/PI double staining followed by flow cytometry. The Q2 and Q3 quadrants represent apoptosis cells. (**E**) bGCs were transfected with pK25-circTEC and/or miR-144-5p mimic, and then the cell cycle was detected and counted by PI binding followed by flow cytometry. (**F**) We transfected bGCs with pK25-circTEC and/or miR-144-5p mimic, and then cell proliferation was measured and counted by EdU assays. Red for EdU and blue for DAPI. The same letter denotes no significant difference (*p*  >  0.05), while different letters indicate a mean significant difference (*p*  <  0.05). ** *p*  <  0.01. Scale bars: 200 μm.

To investigate the roles of miR-144-5p in bGCs, miRNA mimics/inhibitor were synthesized for gain- and loss-of-function analysis. The results of flow cytometry detection showed that the addition of miR-144-5p mimics significantly increased the apoptosis rate of bGCs (Figure 6D). By contrast, miR-144-5p inhibitors significantly decreased the bGCs apoptosis rate (Figure 6A). Moreover, EdU staining analysis discovered that miR-144-5p mimics decreased the proportion of EdU positive cells, and the miR-144-5p inhibitor increased the rate of EdU-positive cells (Figure 6C,F). Cell cycle analysis found that miR-144-5p mimics significantly decreased the cell proliferation index (S phase + G2/M phase) of bGCs, while the miR-144-5p inhibitor significantly increased the cell proliferation index (Figure 6B,E). These results indicate that miR-144-5p promotes bGCs apoptosis and inhibits bGCs proliferation. Furthermore, to confirm the involvement of circTEC in the miR-144-5p-mediated regulation of bGCs functions, we transfected bGCs with pK25-circTEC and/or miR-144-5p mimic. As expected, the overexpression of circTEC reversed the negative effects of miR-144-5p mimics on bGCs, including the apoptosis rate, cell proliferation index, and EdU-positive cells rate (Figure 6D–F). Altogether, our findings suggest that circTEC serves as a sponge for miR-144-5p, promotes bGCs proliferation, and inhibits bGCs apoptosis.

### 2.6. FZD3 Is a Target of miR-144-5p

**Figure 7:**
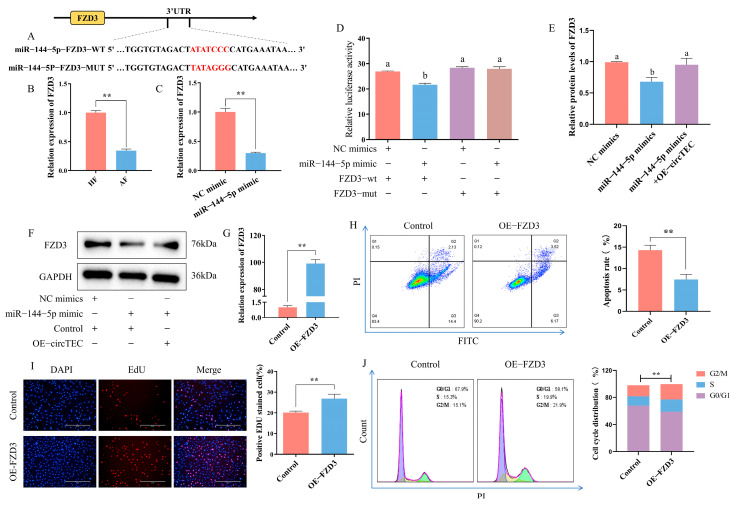
Verification of direct interaction between miR-144-5p and FZD3 and effects of FZD3 on bGCs apoptosis and proliferation. (**A**) Bioinformatics analysis predicted the binding sites between miR-144-5p and FZD3, and the corresponding mutation vector targeting the binding sequences was designed. (**B**) Expression changes of FZD3 in bGCs from HFs and AFs were measured by RT-qPCR. (**C**) Expression of FZD3 was measured by RT-qPCR after miR-144-5p mimics treatment. (**D**) bGCs were co-transfected with miR-144-5p mimics and/or the reporter vector carrying wild-type or mutated corresponding binding site, and then luciferase activity was determined. (**E**,**F**) Protein expression (**F**) and gray value analysis (**E**) of FZD3 in bGCs after transfection of miR-144-5p mimic and/or pK25-circTEC. (**G**) The efficiency of FZD3 overexpression was measured by RT-qPCR. (**H**) The apoptosis rate of bGCs was determined and counted by Annexin V-FITC/PI double staining followed by flow cytometry after the transfection of pEGFP-FZD3. The Q2 and Q3 quadrants represent apoptosis cells. (**I**) The cell proliferation of bGCs was measured and counted by EdU assays after the transfection of pEGFP-FZD3. Red for EdU and blue for DAPI. (**J**) Cell cycle of bGCs was detected and counted by PI binding followed by flow cytometry after the transfection of pEGFP-FZD3. The same letter denotes no significant difference (*p*  >  0.05), while different letters indicate a mean significant difference (*p*  <  0.05). ** *p*  <  0.01. Scale bars: 200 μm.

TargetScan release 7.2 (http://www.targetscan.org/vert_72/, accessed on 6 February 2020) was adopted to predict the potential target gene of miR-144-5p, and FZD3 was highlighted. That is, FZD3 was screened as a possible downstream effector gene of the circTEC–miR-144-5p axis (Figure 7A). At present, it has been confirmed that FZD3 is involved in follicle development [25]. The mRNA expression change of FZD3 in HFs and AFs was verified via RT-qPCR, and the expression level was significantly lower in AF, which was contrary to the expression changes of miR-144-5p during follicular atresia (Figure 7B). To confirm the direct interaction between miR-144-5p and FZD3, we constructed dual luciferase reporter vectors with wild-type or mutated FZD3 3′UTR binding site for miR-144-5p. According to the results, the introduction of miR-144-5p mimics significantly inhibited the activity of the wild-type FZD3 3′UTR vector but had no difference effect on the mutated FZD3 3′UTR vector (Figure 7D). RT-qPCR and WB analysis revealed that miR-144-5p mimics significantly reduced FZD3 mRNA and protein levels, while the overexpression of circTEC reversed the effects of miR-144-5p mimics on the protein expression of FZD3 in bGCs (Figure 7C,E,F). These data indicate that FZD3 directly bonds to miR-144-5p and can be regulated by miR-144-5p and circTEC.

In addition, we further investigated the role of FZD3 in the apoptosis and proliferation of bGCs. The results of flow cytometry detection showed that the overexpression of FZD3 significantly decreased the apoptosis rate of bGCs (Figure 7H). EdU staining analysis showed that the overexpression of FZD3 increased the proportion of EdU-positive cells (Figure 7I). Cell cycle analysis found that FZD3 overexpression increased the proportion of bGCs in the S- and G2/M-phases and decreased the number of G0/G1 phase cells (Figure 7J). These findings suggest that FZD3 can promote bGCs proliferation and inhibit bGCs apoptosis.

## 3. Discussion

CircRNA is known to be highly abundant in the mammalian ovarian follicle, and it has been proven to act as another class of important modulators of various female reproductive physiological or pathological processes. Due to the particularity of reproductive-related cells, the exact mechanism by which circRNA regulates follicular cell function remains unclear. At the same time, due to the difficulty and accuracy of isolating and identifying healthy and atresia follicles, the role and mechanism of circRNA in the regulation of follicular atresia are few and far between [19,23]. Therefore, based on our previous studies that accurately identified healthy and atretic follicles [24,26], we profiled the expression of circRNAs in bGCs obtained from healthy and atretic antral follicles by RNA-Seq for the first time, and a total of 6443 circRNAs from diverse genomic locations were identified, of which 112 were differentially expressed. The abnormal expression of these DEcircRNAs in atretic follicles may be one of the potential factors regulating follicular atresia in buffalo. In addition, the current methods for identifying mammalian atretic follicles are complex and require the detection of many indicators. CircRNA is considered an ideal physiological or pathological biomarker due to its stable circular structure. Thus, these DEcircRNAs may also provide us with independent or supplementary biomarkers for follicular atresia.

The association and interaction between circRNA and their related host genes has always been of great concern. Increasing studies suggested that the regulation of circRNAs on their corresponding host genes is a critical mechanism for their function [27,28]. CircRNAs are able to regulate the expression of host genes at the transcriptional level, post-transcriptional level, translational level, post-translational level, or by encoding polypeptides, and thus they participate in tumor progression [27]. Moreover, it was found that circRNAs become the preferred gene output when the pre-mRNA processing events of host gene are repressed in cells [29]. Based on our previously RNA-seq data [24], the expression changes of DEcircRNAs corresponding with host genes during buffalo follicular atresia were examined, and the researchers found that only a few host genes showed significant changes, which was consistent with previous researches in pigs. In addition, the pathway analysis of DEcircRNAs host genes showed that only two highlighted pathways were the known regulatory pathways for follicular atresia. In summary, these results agreed with the previous viewpoint that changes in the abundance of circRNAs are largely independent of the general transcription of their host genes [19,30].

Granulosa cells are one of the main follicle cell types. During the development of follicles, granulosa cells can synthesize steroid hormones and growth factors, provide nutrients for oocytes through gap junction, and promote the growth and maturation of oocytes. It has been proved that the apoptosis of granulosa cells was the main cause of follicular atresia, which was regulated by a delicate balance of follicle survival factors and atretogenic factors [1,25,30,31]. At present, the regulatory functions of some circRNAs on granulosa cells have been characterized. For instance, circEGFR was certified to promote the estradiol production and proliferation of mouse ovarian granulosa cells [10]. Circ_0008285 was found to enhance free fatty acid secretion of human ovarian granulosa cells [32]. In addition, Maternal et al. [9] reported that circ_0023942 inhibited the proliferation of human ovarian granulosa cells. In this study, by analyzing sequencing data and validation by qRT-PCR, we screened out circTEC, which is down-regulated in AFs. Functional experiments demonstrated that circTEC expression promoted the proliferation and steroid hormone synthesis of bGCs, and it inhibited bGCs apoptosis, revealing circTEC as a potential survival factor in follicular development.

MiRNAs directly target untranslated regions of mRNA by base pairing to cut them, thereby regulating gene expression at the post-transcriptional level [33]. CircRNAs can serve as efficient miRNA sponges, prevent the degradation of downstream target genes, and affect biological processes [34]. It has been reported that ssc-circINHA-001 guaranteed *INHBA* expression by sponging miR-214-5p, miR-7144-3p, and miR-9830-5p, thus further inhibiting porcine granulosa cell apoptosis and follicular atresia [21]. CircINHA promoted porcine granulosa cell proliferation and inhibited porcine granulosa cell apoptosis via *CTGF* as a competing endogenous RNA that directly bound to miR-10a-5p [20]. In addition, circSLC41A1, as a sponge of miR-9820-5p, inhibits its degradation of *SRSF1* mRNA, thereby inhibiting the apoptosis of porcine granulosa cells [22]. In this study, circTEC was mainly localized to the cytoplasm in bGCs, suggesting that circTEC may play a regulatory role by competing with miRNAs. Further experimental results showed that circTEC could directly bind to miR-144-5p and inhibit its activity.

Studies have shown that miR-144-5p is associated with the apoptosis of granulosa cells induced by copper exposure [35], early folliculogenesis [36] and ovarian insufficiency [37]. Additionally, Yang et al. [38] revealed that miR-144-5p can inhibit the apoptosis of damaged granulosa cells and improves ovarian function in rats following cyclophosphamide treatment. However, the function of miR-144-5p in bGCs has not been reported. Therefore, we performed abundant functional experiments to further confirm the biological role of miR-144-5p, and we found that miR-144-5p promotes bGCs apoptosis and inhibits bGCs proliferation. Furthermore, the overexpression of circTEC could reverse the effects of miR-144-5p on the proliferation and apoptosis of bGCs. The above results suggest that circTEC functions as a ceRNA and mitigates the negative effects of miR-144-5p on bGCs.

FZD3, a member of the Frizzled receptors family, is reported to play important roles in embryonic development, tissue homeostasis and human disease [39]. Previous studies have demonstrated that FZD3 was expressed in granulosa cells, theca cells and oocytes of mouse ovary, and it dynamically changed with the development of the estrus cycle [40]. Additionally, FZD3 expression was significantly up-regulated in the cumulus cells from polycystic ovary syndrome patients, which damage the estrogen synthesis of cumulus cells [25]. These studies all point to FZD3 being related to animal reproduction. However, there is little knowledge regarding the role and regulatory mechanism of FZD3 in follicular atresia. We confirmed that FZD3 directly bonds to miR-144-5p and can be regulated by miR-144-5p and circTEC. Functionally, FZD3 can promote bGCs proliferation and inhibit bGCs apoptosis. In addition, we verified that FZD3 was down-regulated in granulosa cells from AF, and its expression was adversely related to miR-144-5p and consistent with circTEC. Taken together, these findings support the hypothesis that in AF, circTEC binds to miR-144-5p and limits the degradation of FZD3, which promotes the proliferation of bGCs and inhibits bGCs apoptosis, thereby inhibiting follicular atresia in buffalo.

## 4. Materials and Methods

### 4.1. Follicle Collection

Ovaries were obtained from adult buffalo (healthy, non-pregnant) at a local abattoir in Nanning and during 1 h transported to the laboratory in sterilized saline maintained at 38 °C. After rinsing and disinfecting with 75% ethanol, ovaries were washed three times with 37 °C normal saline. Then, individual antral follicles (diameter: 5 mm < n < 8 mm) were dissected from the ovarian tissue with ophthalmic scissors and repeatedly rinsed with 0.1 M phosphate buffer (pH 7.25).

### 4.2. Follicle Classification and Selection

Combined with previous research on the classification of buffalo follicles with different degrees of atresia [26], we developed a reasonable classification standard to divide individual antral follicles into healthy follicles (HFs) and atretic follicles (AFs). Briefly, follicles were divided into HFs and AFs by their appearance, internal morphology, the ratio of estradiol (E2) and progesterone (PROG) concentration in the follicular fluid and granulosa cells apoptosis rate. In this study, we selected 6 follicles that met the above three classification criteria for further study, including HFs (n  =  3) and AF (n  =  3).

### 4.3. RNA-Seq

The preparation and sequencing of RNA samples from the granulosa cell of HFs and AF were performed as described previously [24]. Briefly, total RNA was extracted using Trizol reagent (Life Technologies, Mt Waverley, Australia) according to the manufacturer’s protocol. The protocol was as follows: add 1 mL Trizol reagent to 5–10 × 10^6^ cells and leave for 10 min (room temperature) to fully lyse the cells. After drastic mechanic vibration, add 200 μL of chloroform and centrifuge (12,000× *g*, 4 °C for 15 min). Mix the upper water phase with 500 μL of isopropanol, centrifuge (12,000× *g*, 4 °C for 10 min) for RNA deposition, and then add 1 mL 75% ethanol for RNA washing. RNA quality was estimated with an Agilent 2100 Bioanalyzer (Agilent Technologies, Santa Clara, CA, USA) and RNA Nano 6000 Assay Kit (Agilent Technologies, Santa Clara, CA, USA). Only high-quality RNA samples (concentration ≥ 250 ng/μL, RIN ≥ 7.0, total content ≥ 20 ng) were used to construct the sequencing libraries. Sequencing was conducted on an Illumina NovaSeq 6000 platform (Illumina, San Diego, CA, USA). The RNA-seq data can be found in the GEO repository (accession number: GSE157686). Details of the circRNA-seq data analysis are described in Supplementary Material S1.

### 4.4. RT-qPCR

Total RNA was extracted and qualified by an ND-1000 microspectrophotometer (Thermo Scientific, Karlsruhe, Germany) and agarose electrophoresis. For RNase R treatment, <5 μg total RNA with or without 1–3 U μg^−1^ RNase R was incubated at 37 °C for 20 min. The resulting RNA was reverse-transcribed into cDNA using HiScript^®^Ⅲ RT SuperMix (Vazyme, Nanjing, China) for qPCR. For miRNA quantification, reverse transcription was performed with the miRNA 1st-Strand cDNA Synthesis Kit (Vazyme, Nanjing, China). First, the mixture (2 μL of 5× *g* DNA wiper mix and 10 pg^−1^ μg total RNA from the samples, added with ddH2O to a final volume of 10 μL) was prepared in an RNase-free centrifuge tube, mixed well, and the genomic DNA was removed at 42 °C for 2 min. Then, we mixed the mixture from the previous step with 1 μL of 2 µM Stem loop primer, 2 μL of 10 × RT Mix, 2 μL of HiScript II Enzyme Mix and 5 μL of ddH_2_O, and we carried out the first-strand cDNA synthesis reaction under the following conditions: 25 °C 5 min, 50 °C 15 min, and then 85 °C 5 min. qPCR was performed on the Applied Biosystems 7500 Real-Time PCR System (Applied Biosystems, Carlsbad, CA, USA) using ChamQ Universal SYBR qPCR Master Mix (Vazyme, Nanjing, China). Briefly, we mixed 1 μL of 100 ng/mL template cDNA with 0.4 μL of 10 µM forward primer and reverse primer, 10 μL of 2 × ChamQ Universal SYBR qPCR Master Mix and 8.2 μL of ddH_2_O, and we performed a qPCR reaction according to the following conditions: 95 °C 30 s and then 40 cycles of 95 °C 15 s, and 60 °C 30 s. The 2^−ΔΔCT^ method was used to normalize and determine the RNA level. β-actin was used as the internal control for circRNA and coding genes, U6 was used as the internal control for miRNA. All primers used are shown in Supplementary Material S2: Tables S1 and S2.

### 4.5. PCR Amplification and Sanger Sequencing

Divergent primers and convergent primers were designed and synthesized for circTEC, and PCR amplification reactions were performed using cDNA and gDNA as templates. The reaction mixture (22 μL of GoldenStar^®^ T6 Super PCR Mix, 1 μL of 10 µM forward primer and reverse primer and 1 μL of template gDNA/cDNA) was prepared in an RNase-free centrifuge tube and mixed well. Then, we performed the PCR amplification reaction according to the following conditions: 98 °C 2 min, 98 °C 10 s, 60 °C 15 s, 72 °C 2 s, and then 72 °C 2 min. PCR products were visualized after electrophoresis in 2% ethidium bromide-stained agarose gel. To verify the PCR results, the PCR products amplified by specific divergent primers were purified through a TIANgel DNA Purification kit (TIANGEN, Beijing, China). Direct PCR product Sanger sequencing was performed by Sangon Biotech (Shanghai, China). All primers used are shown in Supplementary Material S2: Table S3.

### 4.6. Fluorescence In Situ Hybridization

According to the instructions of the manufacturer, fluorescence in situ hybridization (FISH) assays were performed using in situ hybridization reagents (Servicebio, Wuhan, China) to observe the location of circTEC in bGCs. Briefly, bGCs were cultured on slides and then fixed in FISH fixative for 30 min at room temperature. Then, cell climbing pieces were incubated with PBS containing 0.2% Triton X-100 at room temperature for 5 min to penetrate the cell membrane followed by incubation with the pre-hybridization solution at 37 °C for 1 h. After pre-hybridization, they were hybridized with a specific Cy3-labeled circTEC probe (Cy3–5′-TGCTTTTCTTATACTCATATTGATCTAAGT-3′-Cy3) at 4 °C overnight in the dark and dyed with 4′, 6-diamidino-2-phenylindole (DAPI) for 5 min at room temperature. All of the above processes need to keep the cells moist. Finally, slides were observed and photographed with a fluorescence microscope (Nikon, Tokyo, Japan).

### 4.7. Vector Construction and Transfection

The full length circTEC was cloned into a pK25ssAAV-ciR vector (Geneseed, Guangzhou, China) at BamH I and EcoR I restriction sites to construct overexpressed plasmid (pK25-circTEC), and the whole length sequence of FZD3 was cloned into the pcDNA3.1 vector (Geneseed, Guangzhou, China) at Xba I and EcoR I restriction sites to obtain overexpressed plasmid (pK25-circTEC). The mimics and inhibitor of miRNA were purchased from RiboBio (Guangzhou, China). All vectors were verified by sequencing. As reported previously [41], bGCs were collected from follicular fluid and cultured in normal DMEM (Gibco-BRL, Grand Island, NE, USA) containing 1% penicillin and streptomycin (HyClone, South Logan, UT, USA) and 10% fetal bovine serum (Gibco-BRL, Grand Island, NE, USA). MiRNA mimics, miRNA inhibitors and overexpression vectors were transfected into bGCs using Lipofectamine 3000 reagent (Invitrogen, Carlsbad, CA, USA) according to the manufacturer’s protocol. The cells were harvested for assays 48 h after transfection. All primers used are shown in Supplementary Material S2: Table S4.

### 4.8. Flow Cytometry to Detect Cell Apoptosis and Cell Cycle

The apoptosis of bGCs was detected using an FITC Annexin V Apoptosis Detection Kit I (BD Pharmingen, San Jose, CA, USA). To be specific, the collected bGCs were washed twice with cold PBS and then resuspended in 1 × binding buffer. Then, cells were incubated with 5 μL FITC Annexin V and 5 μL PI at room temperature for 15 min in the dark. Next, the cell samples were assessed by flow cytometry (ACCURI C6, BD, San Jose, CA, USA) within 1 h, and the experimental data were analyzed using FlowJo V10.6.2 software (BD, San Jose, CA, USA).

The cell cycle of bGCs was measured by the Cell Cycle Staining Kit (Multisciences, Hangzhou, China) according to the manufacturer’s protocol. Briefly, 1 × 10^6^ bGCs from different treatment groups were collected and washed once with PBS and resuspended in a mixture containing 1 mL DNA Staining solution (the components include RNase A and propidium iodide) and 10 μL permeabilization solution. Then, cell suspensions were incubated at room temperature for 30 min in the dark. Finally, the cell samples were assessed by flow cytometry within 1 h, and the experimental data were analyzed using FlowJo V10.6.2 software (BD, San Jose, CA, USA).

### 4.9. Enzyme Linked Immunosorbent Assay

The ability of bGC to synthesize steroid hormones was tested after culturing with 19-hydroxyandrostenedione (0.1 mM, Sigma-Aldrich, St. Louis, MO, USA) in bGC medium. At the same time, in order to avoid the difference in the number of cells in different treatment groups, the cells in the test medium were counted first, and then the hormone concentration was converted according to the cell number proportion. The levels of E2 and PROG in the bGCs cultures were measured using the enzyme linked immunosorbent assay (ELISA) kit (Cusabio, Wuhan, China, CSB-E08893b, CSB-E08172b). Briefly, we added 50 μL of each of the test samples, HRP conjugate, and corresponding antibody to each well in turn, mixed them well, and then incubated for 60 min at 37 °C. Then, we aspirated each well and washed three times with wash buffer. Afterwards, we added 50 μL each of substrate A and substrate B to the well, mixed them well, and incubated them for 15 min at 37 °C in the dark. After we added 50 μL of stop solution to each well, the OD value was detected at 450 nm by an enzyme-labeled instrument (Tecan Trading AG, Mannedorf, Switzerland), and the data were analyzed by ELISAcalc v0.2 software (Multisciences, Hangzhou, China). The intra-assay coefficient of variations did not exceed 15%.

### 4.10. 5-Ethynyl-20-Deoxyuridine (EdU) Assay

The EdU incorporation assay was performed using a Cell-Light EdU DNA cell proliferation kit (RiboBio, Shanghai, China) in the light of the manufacturer’s instructions. Briefly, bGCs seeded in 96-well plates were incubated with 10 µM EdU reagent at 37 °C for 2 h in the dark and then fixed with 4% paraformaldehyde. The Apollo Dye Solution was used to stain proliferating cells, and Hoechst 33,342 was used to stain the nucleic acid in all cells. Finally, images were taken from a fluorescence microscope (Nikon, Tokyo, Japan), and the number of EdU-positive cells was counted by ImageJ 1.53k software (National Institutes of Health, Bethesda, MD, USA).

### 4.11. Western Blotting (WB) Analysis

Total proteins from collected bGCs were extracted with RIPA buffer (Servicebio, Wuhan, China) containing 1% protease inhibitor PMSF (Servicebio, Wuhan, China), and protein concentrations were determined using a BCA protein assay kit (Solarbio, Beijing, China). The proteins were separated via 10% SDS- polyacrylamide gel (Bio-Rad, Hercules, CA, USA) electrophoresis and transferred onto the nitrocellulose membrane (Millipore, Burlington, MA, USA). Then, the membranes were blocked with 5% nonfat milk at room temperature for 2 h and incubated with primary antibodies specific for GAPDH (Proteintech, Wuhan, China) or FZD3 (Bioss, Beijing, China) at 4 °C overnight. After incubation with HRP-conjugated secondary antibody at room temperature for 1 h, the target protein bands were visualized using the ECL Plus (Solarbio, Beijing, China) and the images were generated by the ChemiDoc XRS+ system (Bio-Rad, Hercules, CA, USA). The quantified value of the protein band was calculated using ImageJ 1.53k software (National Institutes of Health, Bethesda, MD, USA).

### 4.12. Dual-Luciferase Reporter Assay

The full-length circTEC, 3′UTR region of FZD3 and their corresponding mutant versions with mutant miR-144-5p and/or miR-29c-5p binding sites were cloned into the luciferase reporter vector pmiRGLO. All these plasmids were constructed by Tsingke Biotech (Beijing, China) and validated by sequencing. Luciferase activity was validated using HEK293T cells. To be specific, 293T cells (5 × 10^4^ per well) were plated on 96-well plates 24 h before transfection and co-transfected with a mixture of 100 ng recombinant reporter vectors, 2 ng Renilla luciferase reporter vectors (pRL-TK) and miRNA mimics at the indicated concentration using X-tremeGENE™ HP DNA Transfection Reagent (Roche, Basel, Switzerland). Then, 48 h after transfection, the Luc-Pair TM Duo-Luciferase ASSay Kit 2.0 (iGeneBio, Guangzhou, China) was used to detect the luciferase activity according to the protocol of the manufacturer.

### 4.13. Statistical Analysis

The statistical analysis was performed using GraphPad Prism 8.0 software (GraphPad Software, San Diego, CA, USA). Statistically significant differences were calculated using Student’s *t*-test and one-way ANOVA as appropriate. All experiments were repeated at least three times independently, and the results are expressed as the mean ± SEM. Unless otherwise stated, differences were considered significant when *p* < 0.05.

## 5. Conclusions

In conclusion, our study provided an overview of the circRNA expression profile in granulosa cells derived from buffalo healthy and atretic follicles. We uncovered a novel circRNA. circTEC was significantly down-regulated in atretic follicles and could effectively promote the proliferation and steroid hormone synthesis of bGCs and inhibit bGCs apoptosis. Mechanistically, circTEC functions as a ceRNA for miR-144-5p to prevent the degradation of FZD3 mRNA and then inhibiting follicular atresia in buffalo. Our results will contribute to further understanding the mechanism of follicular atresia in buffalo and provide a reference for human reproduction studies.

## Data Availability

The datasets presented in this study can be found in online repositories. The names of the repository and accession number can be found in the article.

## Supplementary Materials

The following supporting information can be downloaded at https://www.mdpi.com/article/10.3390/ijms26010153/s1. References [42,43,44,45,46] are cited in the Supplementary Materials.
