# Supplementary material for: CircTEC Inhibits the Follicular Atresia in Buffalo (Bubalus bubalis) via Targeting miR-144-5p/FZD3 Signaling Axis
Source: Int J Mol Sci. 2024 Dec 27;26(1):153. doi: 10.3390/ijms26010153 (PMC11719787; doi:10.3390/ijms26010153)
Supplement: Supplementary file 1 [file ijms-26-00153-s001.zip › Supplementary material S1.pdf]

The circRNA library was based on the lncRNA library as described previously [1].

1. Flow chart of bioinformatics analysis

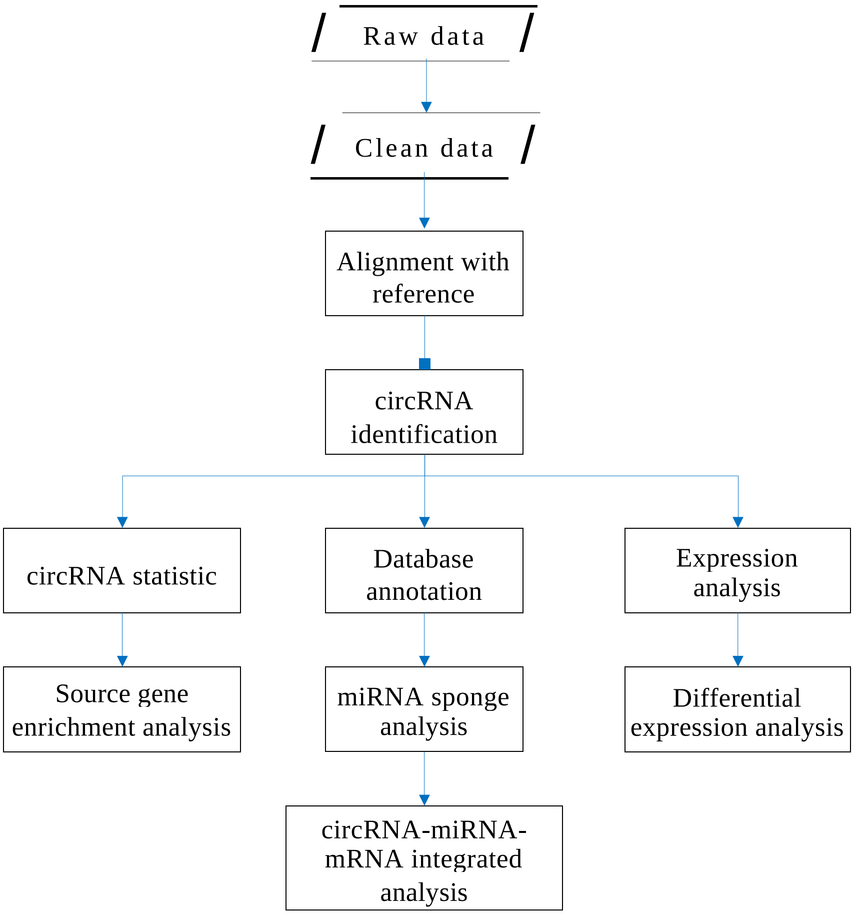

Fig S1. Flow chart of bioinformatics analysis

1.1 Filtering of Clean Reads

Reads obtained from the sequencing machines included raw reads containing adapters or low quality bases which would affect the following analysis. Thus, to get high quality clean reads, reads would be further filtered according to the following rules:

(1) removing reads containing adapters

(2) removing reads containing more than 10% of unknown nucleotides (N);

(3) removing low quality reads containing more than 50% of low quality (Q-value≤20) bases.

1.2 Alignment with Ribosome RNA (rRNA)

Different species and sample qualities would affect the efficiency of experimental ribosome RNA removal. Thus, short reads alignment tool Bowtie2 [2] was used for mapping reads to ribosome RNA (rRNA) database. The rRNA mapped reads will be removed. The remaining reads were further used in alignment and analysis.

1.3 Alignment with Reference Genome

The rRNA removed reads of each sample were then mapped to reference genome by TopHat2 [3] (version 2.0.3.12), respectively. After aligned with reference genome, the reads that could be mapped to the genomes were discarded, and the unmapped reads were then collected for circRNA identification.

1.4 Identification of circRNA

20mers from both ends of the unmapped reads were extracted and aligned to the reference genome to find unique anchor positions within splice site. Anchor reads that aligned in the reversed orientation (head-to tail) indicated circRNA splicing and then were subjected to find_circ[4] to identify circRNAs. The anchor alignments were then extended such that the complete read aligns and the breakpoints were flanked by GU/AG splice sites. A candidate circRNA was called if it was supported by at least two unique back spliced reads at least in one sample.

1.5 circRNA statistics

The identified circRNAs were subjected to statistical analysis of type, chromosome distribution and length distribution.

1.6 Functional enrichment analysis of source gene

Source gene is the origin gene of a circRNA. We performed the functional enrichment analysis of source genes to study the main functions of these source genes of circRNAs.

1.6.1 GO Enrichment Analysis

Gene Ontology (GO) is an international standardized gene functional classification system which offers a dynamic-updated controlled vocabulary and a strictly defined concept to comprehensively describe properties of genes and their products in any organism. GO has three ontologies: molecular function, cellular component and biological process. The basic unit of GO is GO-term. Each GO-term belongs to a type of ontology.

GO enrichment analysis provides all GO terms that significantly enriched in source genes comparing to the genome background, and filter the source genes that correspond to biological functions. Firstly all source genes were mapped to GO terms in the Gene Ontology database (http://www.geneontology.org/), gene numbers were calculated for every term, significantly enriched GO terms in source genes comparing to the genome background were defined by hypergeometric test. The calculating formula of P-value is as follows:

$$P = 1 - \sum_{i=0}^{m-1} \frac{\binom{M}{i}\binom{N-M}{n-i}}{\binom{N}{n}}$$

Here N is the number of all genes with GO annotation; n is the number of source genes in N; M is the number of all genes that are annotated to the certain GO terms; mis the number of source genes in M. The calculated p-value were gone through FDR Correction, taking FDR $\leqslant$ 0.05 as a threshold. GO terms meeting this condition were defined as significantly enriched GO terms in source genes. This analysis was able to recognize the main biological functions that source genes exercise.

1.6.2 Pathway Enrichment Analysis

Genes usually interact with each other to play roles in certain biological functions. Pathway-based analysis helps to further understand genes biological functions. KEGG is the major public pathway-related database[5]. Pathway enrichment analysis identified significantly enriched metabolic pathways or signal transduction pathways in source genes comparing with the whole genome background. The calculating formula is the same as that in GO analysis:

$$P = 1 - \sum_{i=0}^{m-1} \frac{\binom{M}{i}\binom{N-M}{n-i}}{\binom{N}{n}}$$

HereN is the number of all genes that with KEGG annotation,n is the number of source genes in N, M is the number of all genes annotated to specific pathways, and m is number of source genes in M. The calculated p-value was gone through FDR Correction, taking FDR $\leq$ 0.05 as a threshold. Pathways meeting this condition were defined as significantly enriched pathways in source genes.

1.7 Quantification of circRNA Abundance

To quantify circRNAs, back-spliced junction reads were scaled to RPM (Reads Per Million mapped reads), and the formula is shown as follows:

$$\text{RPM}=\frac{10^6 C}{N}$$

In this formula, C is the number of back-spliced junction reads that uniquely aligned to acircRNA. N is the total number of back-spliced junction reads. The RPM method is able to eliminate the influence of different

sequencing data amount on the calculation of circRNA expression. Therefore, the calculated expression can be directly used for comparing the differential expression among samples.

1.8 Analysis of Differentially expressed circRNAs

To identify differentially expressed circRNAs across samples or groups, the edgeR package (http://www.r- project.org/) was used. We identified circRNAs with a fold change ≥2 and a P value <0.05 in a comparison between samples or groups as significant differentially expressed circRNAs.

1.9 Database annotation of circRNAs

CircRNAs were blasted against the circBase[6] for annotation. Those cannot be annotated were defined as novel circRNAs.

2. Reference

1. Pan Y, Yang S, Cheng J, et al. Whole-Transcriptome Analysis of LncRNAs Mediated ceRNA Regulation in Granulosa Cells Isolated From Healthy and Atresia Follicles of Chinese Buffalo. Front Vet Sci. 2021 Jul 14;8:680182.

2. Langmead B, Salzberg S L. Fast gapped-read alignment with Bowtie 2[J]. Nature methods, 2012, 9(4): 357-359.

3. Kim D, Pertea G, Trapnell C, et al. TopHat2: accurate alignment of transcriptomes in the presence of insertions, deletions and gene fusions[J]. Genome biology, 2013, 14(4): R36.

4. Memczak S, Jens M, ElefsiniotiA, et al. Circular RNAs area large class of animal RNAs with regulatory potency[J]. Nature, 2013, 495(7441): 333-338.

5. Kanehisa, M., M. Araki, et al. KEGG for linking genomes to life and the environment. Nucleic Acids Res. 2008.36 (Database issue): D480-4.

6. Glažar, P., Papavasileiou, P. and Rajewsky, N. (2014) circBase: A database for circular RNAs. RNA, 20, 1666-1670
