# Supplementary material for: CircTEC Inhibits the Follicular Atresia in Buffalo (Bubalus bubalis) via Targeting miR-144-5p/FZD3 Signaling Axis
Source: Int J Mol Sci. 2024 Dec 27;26(1):153. doi: 10.3390/ijms26010153 (PMC11719787; doi:10.3390/ijms26010153)
Supplement: Supplementary file 1 [file ijms-26-00153-s001.zip › Supplementary material S2.pdf]

All primer sequences used in this study are shown in Supplementary Material S2.

Table S1. Primers for RT-qPCR.

Name	Forward (5'-3')	Reverse (5'-3')
circTEC	TCCAAGCGACAGAAGCACA	GCTGGTGTTGGAGGTAGTG
Bax	GACACAGACTCTCCCCGA	GAAGGAAGTCCAATGTCCA
Bcl2	GTGGATGACCGAGTACCTGAAC	AGACAGCCAGGAGAAATCAAAC
P53	GTGGATGACCGAGTACCTGAAC	GCACTTCATTCGGACATTCA
Caspase9	CTCGCTTTGGGACGCTCTG	TTTCATGGGTCATCCTGTTTTGC
Caspase3	CAGACAGTGGTGCTGAGGATGA	GCTACCTTTCGGTTAACCCGA
PCNA	GTGAACCTGCAGAGCATGGACTCGT	CGTGTCCGCGTTATCTTCAGCTCTT
CYClinD1	GTCCTGGTGAACAAACT	CGGGTTGGAAATGAACTT
CYP11A1	AATGGCTGGCTTAACCTCTAC	CCACGTCTTCAGGGTGAATT
CYP19A1	TTCCCAAAGAATAATGAGT	GTGACCAGGATGACCTTCA
FZD3	TCTTGTGAGCCTATTACCTTG	CAATGCTGCCGTCTGTTGGT
β-actin	ACCGCAAATGCTTCTAGG	ATCCAACCGACTGCTGTC
miR-144-5p	GCGCGTGGGATATCATCATA	AGTGCAGGGTCCGAGGTATT
miR-29c-5p	GCGCTGATTTCATTTGGTGA	AGTGCAGGGTCCGAGGTATT
U6	CTCGCTTCGGCAGCACA	AACGCTTCACGAATTTGCGT

Table S2. Primers for RT-PCR.

Name	Forward (5'-3')
RT-miR-144-5p	GTCGTATCCAGTGCAGGGTCCGAGGTATTCGCACTGGATACGACACAGTA
RT-miR-29c-5p	GTCGTATCCAGTGCAGGGTCCGAGGTATTCGCACTGGATACGACTCTACG

Table S3. Primers for PCR.

Name	Forward (5'-3')	Reverse (5'-3')
circTEC Divergent primer	CGCCAGAAATAAAGAAACGA	CTGGTGTTGGAGGTAGTGC
circTEC Convergent primer	TCCGCCTGAAGAAGAAAATG	ACTCCCATATTTATCTCTTGC

Table S4. Primers for vector construction.

Name	Forward (5'-3')
circTEC-F	CGGAATTCTAATACTTTCAGCGCCAGAAATAAAGAAACGA
circTEC-R	CGGGATCCAGTTGTTCTTACCTGGTGTTGGAGGTAGTGC
FZD3-F	CGGAATTCATGGCTATGAGTAGGATCGTCTT
FZD3-R	GCTCTAGAAGCACTGGTTCCATCTTCTTCAA
